# Game-Theoretic Decision Support for Cyber Forensic Investigations

**DOI:** 10.3390/s21165300

**Published:** 2021-08-05

**Authors:** Antonia Nisioti, George Loukas, Stefan Rass, Emmanouil Panaousis

**Affiliations:** 1 Department of Computing and Mathematical Sciences, University of Greenwich, London SE10 9BD, UK; a.nisioti@greenwich.ac.uk (A.N.); g.loukas@greenwich.ac.uk (G.L.); 2 Institut of Artificial Intelligence and Cybersecurity, Universitaet Klagenfurt, Universitatsstrasse 65-67, 9020 Klagenfurt, Austria; stefan.rass@aau.at

**Keywords:** cyber forensics, digital forensics, game theory, bayesian game, multi-stage attacks, decision support, optimisation

## Abstract

The use of anti-forensic techniques is a very common practice that stealthy adversaries may deploy to minimise their traces and make the investigation of an incident harder by evading detection and attribution. In this paper, we study the interaction between a cyber forensic Investigator and a strategic Attacker using a game-theoretic framework. This is based on a Bayesian game of incomplete information played on a multi-host cyber forensics investigation graph of actions traversed by both players. The edges of the graph represent players’ actions across different hosts in a network. In alignment with the concept of Bayesian games, we define two Attacker types to represent their ability of deploying anti-forensic techniques to conceal their activities. In this way, our model allows the Investigator to identify the optimal investigating policy taking into consideration the cost and impact of the available actions, while coping with the uncertainty of the Attacker’s type and strategic decisions. To evaluate our model, we construct a realistic case study based on threat reports and data extracted from the MITRE ATT&CK STIX repository, Common Vulnerability Scoring System (CVSS), and interviews with cyber-security practitioners. We use the case study to compare the performance of the proposed method against two other investigative methods and three different types of Attackers.

## 1. Introduction

As adversaries evolve their techniques, both in sophistication and variety, cyber forensics investigations are becoming more complex and time consuming [1]. Modern threats such as Advanced Persistent Threats (APTs) consist of a large number of steps and include a wide variety of Tactics, Techniques, and Procedures (TTPs), which allow adversaries to achieve their goals and avoid detection at the same time.

To address these problems and increase the efficiency of cyber investigations, we previously proposed DISCLOSE, a data-driven decision support framework, which utilises Tactics, Techniques, and Procedures (TTPs) to offer optimal inspections choices to the investigator [2]. To do so, DISCLOSE combines extracted threat intelligence information regarding TTPs with an attack life-cycle model and the progress of the investigation. In this way, it optimises the choices of the investigator taking into consideration the sophistication and diversity of the TTPs used by adversaries.

However, in most cases, such sophisticated and determined attackers will not choose their actions only based on the immediate benefit they may collect, but rather consider the stealthiness of their actions even if this results in the use of extra resources. Specifically, adversaries will additionally deploy anti-forensic TTPs to either conceal part of their trail or to increase the complexity and difficulty of the investigative process, which in turn incurs delays in the investigation [3] and can significantly increase the financial, reputational or other impact of the attack [4,5]. Thus, a decision support system that aims to optimise a cyber forensic investigation process can be benefited from investigating the strategic nature of adversaries.

Motivated by this, we extend our previous work in order to also capture the Attacker’s strategic movements and use of anti-forensic techniques instead of simply modelling the investigator’s movements.

Specifically, this work offers the following original contributions:

- The interaction between the Investigator and a strategic Attacker is modelled using a game theoretic framework. Although game theory has been utilised extensively in the past for modelling the interaction between defending and attacking agents to solve a wide variety of cyber security problems, it has not been used for cyber forensics. In this way, contrary to our previous work, we do not simply assume the actions of the Attacker but rather model his/her motivations and strategies.
- A game-theoretic decision support framework is presented that, similarly to DISCLOSE, aims to increase the efficiency of the investigator against a strategic adversary with anti-forensic capabilities. Efficiency refers to the ratio of acquired benefit from the uncovered attack actions to the consumed cost, such as time and computing resources. Thus, our aim is to increase the collected benefit across a wide range of investigations while decreasing the spending cost. However, contrary to our previous work and the literature, we explicitly model the anti-forensic capabilities of the Attacker using different Bayesian types and incomplete information on the side of the Investigator.

This paper is a first step towards (i) use game-theoretic concepts for the optimisation of multi-stage cyber forensic investigations and (ii) model a cyber forensic investigation as a set of incomplete information games that takes in consideration the anti-forensic abilities of the Attacker.

To achieve the aforementioned, we use a predefined graph consisting of edges that represent real TTPs across different hosts of the network and nodes that represent decision points, where each game takes place. The use of a graph of TTPs aligns with the multi-stage nature of modern sophisticated cyber incidents, such as APTs. To capture the strategic nature of the Attacker and his/her ability to use anti-forensic TTPs, we use the concept of Bayesian games of incomplete information. We allow two Attacker types per decision point, one that applies an anti-forensic technique to conceal his/her activities and one that does not, and assume a probabilistic distribution calculated from past incident reports based on which the types are drawn. This asymmetry in the provided information, i.e., the need of the Investigator to choose a TTP without the prior knowledge of the Attacker’s type, models the uncertainty of the Investigator in a real-world case.

We evaluate the proposed method against two other investigative methods using a case study that consists of a graph of attack actions on multiple hosts created using real-world data gathered through:

- The MITRE ATT&CK Structured Threat Information Expression (STIX) [6] repository, a well-known threat intelligence knowledge base that contains structured information on adversarial TTPs from real incident reports;
- The Common Vulnerability Scoring System (CVSS);
- Interviews with six cyber-security practitioners.

The rest of the paper is organised as follows. Section 2 presents the literature that is most closely related to ours, which includes (i) game-theoretic concepts for the support of cyber forensic investigations or defence against APTs and (ii) non-game theoretic approaches for the optimisation of such investigations. Section 3 presents the environment where the proposed game-theoretic model can be realised as well as the building components and the solution of the game itself. Section 4 presents a case study of a graph, consisting of a set of real adversarial TTPs, which is used for the evaluation of the proposed game-theoretic method against two other investigative methods as well as a comparative discussion of their performances. Finally, Section 6 concludes the paper and discusses future work.

## 2. Related Work

Although game theory has been utilised in many sub-fields of cyber-security, such as privacy, honeypot allocation, DDoS defence etc., works that investigate the use of game theory for the forensic analysis of multi-stage cyber attacks are non-existent. Thus, in this section, we present works that can be classified into the two following categories: (i) game-theoretic methods for cyber forensics, (ii) non-game-theoretic decision support for cyber forensics and (iii) game-theoretic methods against APTs. We have chosen to include bibliography related to APTs as they reflect the strategic nature of the Attacker that we assume in this paper. Moreover, a forensic investigation of an incident undertaken by an APT is the most interesting case for us making the application of game theory more valuable than in the cases of commodity attacks, which are far less strategic. For a more detailed review of the game-theoretic literature in the cyber-security field, the reader may refer to [7,8].

### 2.1. Game Theory for Cyber Forensics

Hasanabadi et al. [9] focus on rootkit defence by using characteristics for both real rootkit and anti-rootkit tools to formulate the interaction of an Attacker and a Defender as a non-zero sum game. The game between the two players is simulated as a Fictitious Play, i.e., it utilises the empirical frequency of the opponent’s moves, and allows for the identification of the most stable defence strategies but also the most destructive adversarial strategies. Similarly, the authors of [10] propose a Nash game-theoretic approach for tool selection to allow investigators to optimally choose amongst a wide range of available forensic tools. The proposed method takes in consideration the priorities and characteristics of the case, such as time and file sensitivity, and produces an optimal tool choice. Although both of the aforementioned works focus on assisting the decision process during the technical part of an investigation, similarly to the proposed solution, our work does not focus on the selection of the right tool, but rather on the selection of the adversarial activities that may have taken place.

Yan et al. [11] propose a Stackelberg zero-sum game between an Auditor and an Attacker to increase the efficiency of database alert auditing prioritisation. Similarly to our work, the authors aim to optimise the auditing process of the analyst taking in consideration the strategic nature of the opponent. However, while they treat the auditing process of all available alerts as a single unit, we choose to break down the investigation in steps in order take in consideration the multi-stage nature of modern cyber-incidents. Finally, Stamm et al. in [12,13] model the interaction between an investigator and an adversary with anti-forensic capabilities as zero-sum game. Although, our work also assumes an attacker with the capability manipulating the evidence after an attack, Stamm et al.’s work focuses on digital video and image forgery. Thus, while the authors propose a game-theoretic method to help the investigator detect the use of anti-forensic on multimedia evidence, we aim to optimise the attack actions the defender selects to investigate taking in consideration the potential use of anti-forensic techniques by the attacker on multi-stage cyber incidents.

### 2.2. Non-Game-Theoretic Decision Support

Horsman et al. [14] propose the Case-Based Reasoning Forensic Triager (CBR-FT), which aims to increase the efficiency of the triage process of the investigation. CBR-FT uses a past incident knowledge base similarly to our approach, which has to be populated manually by investigators, to produce a list with the most popular paths of a file system to contain evidence. Contrary to our work, CBR-FT does not take in consideration the multi step nature of cyber incidents or the available actions of the adversary but instead uses file system paths, which limits the applicability of the proposed method. Similarly to our work, De Braekt et al. [15] aim to increase the efficiency of a cyber forensic investigation taking in consideration the cost of the analysis to provide guidelines for the optimisation of the workload and the usage of the available resources. We also have the same goal but we achieve this in a different way by using TTPs to model multistage incidents on a graph and game theory to identify the optimal choices for the Investigator.

A non-game-theoretic decision support framework, DISCLOSE, for multistage forensic investigations is proposed by Nisioti et al. in [2]. Similarly to the proposed framework, DISCLOSE models the investigation as a collection of steps using adversarial TTPs and considers both the cost and benefit of each TTP to provide optimal suggestions to the investigator. However, contrary to this work, DISCLOSE does not model the Attacker but solely the Investigator and thus is also not capable of taking in consideration the use of anti-forensic techniques.

Finally, there are a number of works that utilise graphs, as we do, to capture the multi-step nature of cyber incidents. Liu et al. [16,17,18] propose the use of evidence graphs to support forensic investigations, while Barrère et al. [19] introduce core evidence graphs, which are a condensed version of attack graphs, to increase the efficiency of an investigation. However, all of those works utilise vulnerabilities as the building block of the graph. Instead of this, we propose the use of TTPs as they align with the current threat landscape and allow the application of the proposed method in a wider range of investigations. One commonality of the aforementioned works in this subsection is that they do not model the adversary or take in consideration its strategic nature. On the contrary, our approach utilises game theory to model a strategic sophisticated attacker and, specifically, a Bayesian game to represent the attackers ability to use anti-forensic techniques.

### 2.3. Game Theory against APT

In the literature, two of the most similar works to our approach are presented by Zhu and Rass and Rass et al. in [20,21], correspondingly. In the first work, the authors choose to divide an APT in three main phases: Initial penetration and establishment, Learning and propagation and Damage and define the whole process as a multi-phase multistage (MPMS) game. They then model a separate phase as a different type of game, where the first phase is a Bayesian game, the second is a sequential Nash game running on a subnet graph and, finally, the third phase is a static Nash game. In the second work, the interaction between a stealthy APT adversary is modelled as a Bayesian zero sum game on a predefined graph by allowing different types for the Attacker to represent the uncertainty of the Defender regarding the entry point of the attacker.

Even though there are many similarities between these works and our approach, such as the use a Bayesian game as well as the use of a predefined graph to model a multi-step APT incident, there are also three significant differences. Firstly, we allow the types of the Attacker to represent the uncertainty about the use of anti-forensics by the Attacker. Secondly, we focus on the post mortem investigation of the incident instead of its prevention and, thus, our goal is to assist the investigator to reveal the performed attack actions in the most efficient way. Thirdly, while [21] uses vulnerabilities as the building component of the graph and [20] divides the incident in three phases, we choose to use adversarial TTPs, as they allow for a better representation of a modern cyber incident and greater applicability across a wide range of cases [2].

Finally, another Bayesian game against APTs, which similarly to our work, considers the multi-stage and deceptive nature of APTs is presented by Huang et al. [22]. Specifically, the authors propose an online game for proactive APT defence, where the Defender dynamically develops a belief regarding the Attacker based on the observed actions and uses it to enhance the defences of the infrastructure in order to mitigate future attacks. As before, the main difference between this work and our work is the focus on the forensic investigation instead of the defence problem as well as the inclusion of the anti-forensic capabilities of the Attacker, but also the lack of visible signals in our approach, which models realistically the inability of the investigator to observe the Attacker.

## 3. Game-Theoretic Decision Support for Optimising Cyber Forensic Investigations

A cyber forensics investigator, henceforth referred to as the Investigator, is commissioned with the task of disclosing evidence related to an incurred cyber incident. The choices of the Investigator, akin to *inspection actions*, are represented by the space of *attack actions*, which are available to the Attacker during the attack. More specifically, at any point of the investigation process while on a host of the network, the Investigator faces the challenge of selecting the next inspection action to undertake. To find optimal strategies for this cyber forensics challenge, we apply two game-theoretic concepts that capture the asynchronous interactions between an Attacker and the Investigator.

We firstly define the Nash Cyber Investigation Game (NCIG), derive the Nash Equilibrium (NE), and discuss how this can be translated to actionable advice for the Investigator. We go beyond NCIG by considering different Attacker types, in order to capture situations where the Attacker wishes to remain stealthy by minimising his/her traces during the forensic investigation. Thus, we model the Bayesian Cyber Investigation Game (BCIG) as a Bayesian game with two different Attacker types based on whether he/she deploys *anti-forensics techniques* to decrease the likelihood of detection and attribution.

### 3.1. Environment

Let us assume that there is an Attacker that aims to infiltrate a corporate network with the aim of reaching a predefined target. To accomplish this, the Attacker performs a set of attack actions, which collectively can be referred to as a cyber incident. The set of available attack actions at each step of the attack depends on the network structure, technologies in use, current defence mechanisms and other characteristics of the targeted organisation. After the completion of the incident, i.e., after the Attacker has already accomplished his/her goal, an Investigator is called to uncover the performed actions. This leads to a standard forensic process, where the Investigator aims to disclose the desirable forensic evidence in a way that optimises how he/she chooses forensic actions. We assume that each of these forensic actions is capable of revealing an attack action, as we have also proposed in [2].

Every attack and inspection action takes place on a host of a network, which can be any physical or virtual device. Most likely, the incident under investigation may include more than one host and more than one action per host. Simply speaking, the Attacker decides which of the available actions will allow him/her to move through the network and reach his/her objective (e.g., perform a specific action on a targeted asset) and the Investigator, who naturally becomes part of the ecosystem after an incident has been suspected, decides which actions will gather appropriate forensic evidence towards uncovering the steps followed by the Attacker. In this way, the organisation will be able to assess the extent of the incident thus strengthening its defence, plan about system recovery, present to an external auditor (e.g., an insurer) facts from the incident, and potentially identify the Attacker.

To demonstrate the above interactions, we define two game-theoretic models, where the Attacker and the Investigator are seen as the strategic rational players who have to choose among a set of attack actions and a set of inspection actions, respectively. This aligns with the characteristics of: (i) modern Attackers, such as APT threat groups, who are highly sophisticated, plan and realise targeted attacks, and (ii) Investigators, who choose their actions based on reasoning and the uncovered evidence. We define a *decision point* in time, when players decide their next action respectively. For the Attacker, a decision point is a state of the attack where he/she chooses his/her next attack action. Similarly, for the Investigator, a decision point is a state of the investigation when he/she has to select the next inspection action. For instance, a decision point for the Attacker would be when he/she is at a specific compromised workstation where he/she has elevated his/her privileges and is now able to choose amongst a set of actions that require admin privileges.

Let the targeted infrastructure, where the players move, be represented in a form of a graph (also referred to as the *investigation graph*), in which the nodes represent decision points, and the edges are available actions to the players. Players move from one decision point (i.e., node) to another by traversing the edges that correspond to the actions they have chosen at each decision point. Due to the nature of their interaction, in our cyber forensic games, the Attacker completes all the actions until an alert has triggered the organisation to commence an investigation. Then, the Investigator will conduct a postmortem analysis of the incident by choosing inspection actions. This is when the Investigator has to disclose the required evidence by starting from a node (e.g., a performed attack action on a server) which was flagged by the detection capabilities of the organisation. Naturally, the Attacker wishes to choose the attack actions that will bring him/her from an *initial node* (e.g., entry point in the attack surface of the organisation’s infrastructure) to an *end node*, which is where he/she will collect his/her reward. As explained, each node is a point where both the Attacker and the Investigator choose their next action.

Let E be the set of available edges for players to traverse at a decision point on the graph. Each edge is an attack action on a specific host of the network that the Attacker can use to move from one node to another and the Investigator may investigate. For instance, an attack action is the creation of a scheduled task, while an inspection action is the one that uncovers evidence of this malicious activity. Let each edge $e_{i}\in E$ have a benefit $\beta_{i}$ for both players, which in this paper is common knowledge to both players and represents the negative (resp. positive) impact that an attack action causes to the targeted organisation if performed by the attacker (resp. uncovered by the Investigator). Furthermore, we assume that each attack action represented by $e_{i}$ incurs the Attacker a cost $\alpha_{i}$ while the effort of the Investigator to disclose this action costs him/her $\kappa_{i}$. The cost $\alpha_{i}$ represents the resources that the Attacker spends to undertake this attack action, and likewise, $\kappa_{i}$ refers to the resources that the Investigator spends for investigating this action. In both cases, we allow the cost to represent the time that the player has to spent to either undertake or investigate the attack action. Table 1 summarises our notation.

### 3.2. Nash Cyber Investigation Game

For every decision point, represented by a node in a graph, we formulate a two-player, deterministic, complete-information game, entitled NCIG and denoted by Γ, between the Attacker and the Investigator. At each decision point (i.e., node on the graph), each player chooses their next action from a set of available edges E. In Γ, the players choose without observing each other’s choice but knowing the available actions and utilities of the opponent. Thus, at each decision point, a Nash Cyber Investigation Game is played, where the Attacker has to probabilistically select one of the available attack actions to penetrate the organisation’s defences and the Investigator has to select its inspection strategy to collect the best possible evidence. We assume that for each potential attack action, the Investigator chooses the probability of investigating it.

#### 3.2.1. Strategy Spaces

The normal form of this game is described as follows. At a node of the investigation graph, a pure strategy of the Attacker is to choose an edge $e_{i}$ out of E, towards the exploitation of his/her desirable target. Likewise, the Investigator must choose among these edges when he/she is at the same node, while he/she is conducting the investigation process. Thus, the set of pure strategies of both players consists of all edges in E. In Γ, a pure strategy profile is a pair of actions $\left(e_{i},e_{j}\right),e_{i},e_{j}\in E$ of Attacker and Investigator, giving a pure strategy space of |E|×|E|. For the rest of the paper, the convention is adopted where the Attacker is the row player and the Investigator is the column player.

The *mixed strategy* ρ of the Investigator is a probability distribution over the different edges $e_{i}\in E$ (i.e., pure strategies), where $\rho (e_{i})$ is the probability of choosing $e_{i}$ under mixed strategy ρ. We refer to a mixed strategy of the Investigator as a Randomised Forensic Investigation Plan (RFIP).

Likewise, the Attacker’s *mixed strategy* is a probability distribution α over all available attack actions, where $\alpha (e_{i})$ is the probability of choosing the attack action associated with edge $e_{i}$ under α. We will refer to a mixed strategy of the Attacker as a Randomised Attack Plan (RAP). The use of mixed strategies by the Attacker allows him/her to increase the uncertainty of the Investigator about his/her action choices. On the other hand, the use of mixed strategies by the Investigator allows for the optimisation of the performance of the Investigator across a collections of investigations given that use of mixed strategies by the Attacker.

For the finite set E, we define Π as the set of all probability distributions over it. Since both players have the same strategy space, the set of all mixed strategies for both players is

(1) $$\Pi =\{\rho \left(e_{j}\right)\in R^{+}|\sum_{e_{j}\in E}\rho \left(e_{j}\right)=1\}$$

Therefore, the set of mixed strategy profiles for NCIG is Π×Π.

#### 3.2.2. Expected Payoffs

Next, we formalise the players’ objectives through defining what the probabilities of certain outcomes are and what payoff values each player gains from these outcomes. Each player’s preferences are specified by his/her payoff function, and we define as $U_{A}:\left(e_{i},e_{j}\right)\to R$ and $U_{I}:\left(e_{i},e_{j}\right)\to R$ the payoff functions of the Attacker and Investigator, respectively, when the pure strategy profile $\left(e_{i},e_{j}\right)$ is played. We use the benefit and cost values defines in Section 3.1 to define the players’ payoff functions.

Whenever players choose the same edge (i.e., $e_{i}=e_{j}$), the Investigator collects the benefit $\beta_{i}$ as he/she has uncovered the Attacker’s action and both players pay their respective costs $\alpha_{i}$ and $\kappa_{i}$. On the other hand, if players choose different actions, the Attacker collects the benefit $\beta_{i}$ of not being traced and they both pay their action’s costs.

Formally,

(2) $$U_{A}\left(e_{i},e_{j}\right)=\left\{\begin{array}{cc} -\alpha_{i}, & e_{i}=e_{j}\\ \beta_{i}-\alpha_{i}, & e_{i}\neq e_{j} \end{array}\right.$$

and

(3) $$U_{I}\left(e_{i},e_{j}\right)=\left\{\begin{array}{cc} \beta_{i}-\kappa_{i}, & e_{i}=e_{j}\\ -\kappa_{i}, & e_{i}\neq e_{j} \end{array}\right.$$

As discussed above, Γ is a game per node in the investigation graph. To derive optimal strategies for the Investigator during the repetitions of NCIGs, we deploy the notion of mixed strategies. As players act independently, we enlarge their strategy spaces, to allow them to base their decisions on the outcome of random events that create uncertainty to the opponent about individual strategic choices maximising their payoffs. Hence, both Attacker and Investigator deploy randomised (i.e., mixed) strategies.

We can now define the NCIG as $\Gamma =\langle \left(Attacker,Investigator\right),\Pi \times \Pi ,\left(U_{A},U_{I}\right)\rangle$. For a given mixed strategy (α,ρ), we denote by $U_{A}\left(\alpha ,\rho \right)$ and $U_{I}\left(\alpha ,\rho \right)$ the expected payoffs for the Attacker and the Defender, respectively.

Formally,

(4) $$U_{A}\left(\alpha ,\rho \right)=\sum_{e_{i}\in E}\sum_{e_{j}\in E}U_{A}\left(e_{i},e_{j}\right)\alpha \left(e_{i}\right)\rho \left(e_{j}\right)$$

and

(5) $$U_{I}\left(\alpha ,\rho \right)=\sum_{e_{i}\in E}\sum_{e_{j}\in E}U_{I}\left(e_{i},e_{j}\right)\alpha \left(e_{i}\right)\rho \left(e_{j}\right).$$

#### 3.2.3. Solution

Assuming that both Attacker and Investigator are rational players, their aim is to maximise their expected utilities taking in consideration the other player’s best response.

**Definition** **1.** ***(NE for NCIG).*** *Thus, for a pair of mixed strategies* (α,ρ) *to be a Nash Equilibrium (NE), the following must be satisfied:*

- *The Attacker plays a RAP **α** that is a best-response s.t.*$\alpha \in {\mathrm{argmax}}_{\alpha^{*}\in \Pi }U_{A}\left(\alpha^{*},\rho \right)$;
- *The Defender plays a RFIP **ρ** that is a best-response, which we will refer as the Investigator’s Optimal Randomised Plan (IRP), s.t.*$\rho \in {\mathrm{argmax}}_{\rho^{*}\in \Pi }U_{I}\left(\alpha ,\rho^{*}\right)$.

As this is a bimatrix non-zero-sum game, its solution can be calculated by formulating it as a *linear complementarity problem* (LCP) [23] and solving it using the Lemke–Howson algorithm, as follows:

(6) $$\begin{array}{c} \sum_{e_{i}\in E}U_{I}\left(e_{i},e_{j}\right)\cdot \alpha \left(e_{i}\right)+r_{I}\left(e_{j}\right)=U_{I}^{*},\;\;\forall e_{j}\in E\\ \sum_{e_{j}\in E}U_{A}\left(e_{i},e_{j}\right)\cdot \rho \left(e_{j}\right)+r_{A}\left(e_{i}\right)=U_{A}^{*},\;\;\forall e_{i}\in E\\ \sum_{e_{i}\in E}\alpha \left(e_{i}\right)=1\;\mathrm{and}\;\sum_{e_{j}\in E}\rho \left(e_{j}\right)=1\\ \alpha \left(e_{i}\right)\geq 0\;\mathrm{and}\;\rho \left(e_{j}\right)\geq 0\;\;\forall e_{j},e_{i}\in E\\ r_{A}\left(e_{i}\right)\geq 0\;\mathrm{and}\;r_{I}\left(e_{j}\right)\geq 0\;\;\forall e_{j},e_{i}\in E\\ \alpha \left(e_{i}\right)\cdot r_{A}\left(e_{i}\right)\geq 0\;\mathrm{and}\;\rho \left(e_{j}\right)\cdot r_{I}\left(e_{j}\right)\geq 0\;\;\forall e_{j},e_{i}\in E \end{array}$$

where $r_{I},r_{A}$ are the slack variables as per [23].

#### 3.2.4. Toy Example

Let us now present a toy example of NCIG, where the action space for both players is $E=\{e_{1},e_{2}\}$ and the corresponding cost and benefit values are: $\alpha_{1}=\kappa_{1}=8,\beta_{1}=70,\alpha_{2}=\kappa_{2}=7$ and $\beta_{2}=60$.

The values used in this example for the benefit and cost parameters are not meant to be realistic, but are simply used for demonstration and illustration purposes. On the contrary, the parameters’ values used in Case Study Section, which aims to evaluate and compare the proposed method, are representative of the reality as explained in Section 4.2 and Section 4.3.

The payoffs for the game can be calculated using Formulas (2) and (3) and are shown in Table 2. The pure strategies of the game are easily observable to be $\left\{e_{1},e_{2}\right\}$ and $\left\{e_{2},e_{1}\right\}$. The Nash Equilibrium for mixed strategies of this game is α={0.46923077,0.53076923} and ρ={0.53076923,0.46923077}. This means that the IRP is to choose $e_{1}$ with probability 0.53076923 and $e_{2}$ with probability 0.46923077.

### 3.3. Bayesian Cyber Investigation Game

As aforementioned, in order to approach more accurately the real-world interaction between the Attacker and the Investigation, we define a Bayesian game, entitled Bayesian Cyber Investigation Game (BCIG) and denoted by $\Gamma_{B}$. Building upon NCIG, in BCIG, we assume two different Attacker types θ∈Θ and we let p:θ→[0,1] be the probability distribution representing the Investigator’s belief about the attacker’s type. In BCIG, the Attacker has complete information about his/her type while the Investigator is uncertain of it. Thus, the Investigator receives no signal that can be used to enrich his/her initial knowledge about the Attacker’s type.

Type $\theta_{1}$ is identical with the Attacker from NCIG, i.e., chooses edges $e_{i}$ out of E as his/her actions. Similarly, the second Attacker type $\theta_{2}$ also chooses edges $e_{i}$ as his/her actions but also applies an *anti-forensic technique* to the chosen action. An anti-forensic technique refers to evasion techniques that can be used by attackers to decrease the likelihood of detection, such as encryption or log deletion [24]. In this work, we will assume that there is one possible anti-forensic technique for each edge $e_{i}$ for simplicity reasons, but this assumption could be easily dropped by assuming more than two Attacker types.

As expected, the usage of an anti-forensic technique increases all the constant values associated with each edge by $\beta^{*}$, $\alpha^{*}$ and $\kappa^{*}$, respectively. This is because an anti-forensic technique will obscure the Attacker’s actions, thus increasing the difficulty of the analysis for investigator and decreases the likelihood of detection but also requires extra resources for the attacker. Similarly, uncovering an edge that includes an anti-forensic technique offers extra benefit to the Investigator but also requires more analysis resources. Here, it should be noted, that these values are not the same for each edge as the same anti-forensic technique cannot be combined with all available edges at each decision point.

#### 3.3.1. Strategy Spaces

The pure strategy space for the Investigator in BCIG is the same as in NCIG, i.e., it consists of all edges in E as there is only one type of Investigator. However, there are two types θ for the Attacker and, thus, a pure strategy for him/her is not a single edge in E anymore but a pair of edges, one for each type θ, i.e., $e_{i},e_{j}:\theta \to E$.

Similarly, a mixed strategy RFIP ρ for the Investigator remains the same, with $\rho (e_{i})$ being the probability of $e_{i}$ under ρ. On the contrary, a mixed strategy RAP α for the attacker is now a probability distribution over actions in E for each type θ, i.e. α:θ→Δ(E), with $\alpha (e_{i}|\theta )$ denoting the probability of choosing $e_{i}$ under the mixed strategy α given that the Attacker’s type is θ.

#### 3.3.2. Expected Payoffs

We now revise the payoff functions (2) and (3) for a pair of edges $\left(e_{i},e_{j}\right)$ presented in Section 3.2.2 for the BCIG to include the type θ of the Attacker s.t. $U_{A}:\left(e_{i},e_{j},\theta \right)\to R$ and $U_{I}:\left(e_{i},e_{j},\theta \right)\to R$. When $\theta =\theta_{1}$, the payoff functions for both player’s remain unchanged. However, when $\theta =\theta_{2}$, the benefit and cost values for both players are increased by $\beta^{*}$, $\alpha^{*}$ and $\kappa^{*}$, respectively, when appropriate.

Formally,

(7) $$U_{A}\left(e_{i},e_{j},\theta \right)=\left\{\begin{array}{cc} -\alpha_{i}, & e_{i}=e_{j}\;\mathrm{and}\;\theta =\theta_{1}\\ \beta_{i}-\alpha_{i}, & e_{i}\neq e_{j}\;\mathrm{and}\;\theta =\theta_{1}\\ -\alpha_{i}-\alpha^{*}, & e_{i}=e_{j}\;\mathrm{and}\;\theta =\theta_{2}\\ \beta_{i}+\beta^{*}-\alpha_{i}-\alpha^{*}, & e_{i}\neq e_{j}\;\mathrm{and}\;\theta =\theta_{2} \end{array}\right.$$

and

(8) $$U_{I}\left(e_{i},e_{j},\theta \right)=\left\{\begin{array}{cc} \beta_{i}-\kappa_{i}, & e_{i}=e_{j}\;\mathrm{and}\;\theta =\theta_{1}\\ -\kappa_{i}, & e_{i}\neq e_{j}\;\mathrm{and}\;\theta =\theta_{1}\\ \beta_{i}+\beta^{*}-\kappa_{i}-\kappa^{*}, & e_{i}=e_{j}\;\mathrm{and}\;\theta =\theta_{2}\\ -\kappa_{i}-\kappa^{*}, & e_{i}\neq e_{j}\;\mathrm{and}\;\theta =\theta_{2} \end{array}\right.$$

Contrary to NCIG, where the information known to the players was symmetric, in BCIG there is an asymmetry of information between the Attacker and the Investigator, as the Attacker is aware of his/her type but the Investigator is not. This is reflected to the expected payoff functions for mixed strategies, as the Investigator needs to take in consideration his/her *common belief* about the type of the Attacker in order to cope with his/her uncertainty, while the Attacker is able to use his/her private type information. Formally,

(9) $$U_{A}\left(\alpha ,\rho |\theta \right)=\sum_{e_{i}\in E}\sum_{e_{j}\in E}U_{A}\left(e_{i},e_{j},\theta \right)\alpha \left(e_{i}|\theta \right)\rho \left(e_{j}\right)$$

and

(10) $$U_{I}\left(\alpha ,\rho \right)=\sum_{\theta \in \Theta }p\left(\theta \right)\sum_{e_{i}\in E}\sum_{e_{j}\in E}U_{I}\left(e_{i},e_{j},\theta \right)\alpha \left(e_{i}|\theta \right)\rho \left(e_{j}\right).$$

#### 3.3.3. Solution

**Definition** **2.** ***(BNE in BCIG).*** *As in NCIG, for a pair of mixed strategies* (α,ρ) *to be a Bayesian Nash Equilibrium (BNE), both players have to play their best response taking in consideration the other’s players strategy but also their type (in the case of the Attacker). Formally,* (α,ρ) *is a BNE if the following are satisfied:*

- *The Attacker plays an RAP **α** that is a best-response s.t.*$\alpha \in {\mathrm{argmax}}_{\alpha^{*}\in \Pi }U_{A}\left(\alpha^{*},\rho |\theta \right),\forall \theta \in \Theta$;
- *The Defender plays an RFIP **ρ**, which we refer as IRP, that is a best-response s.t.*$\rho \in {\mathrm{argmax}}_{\rho^{*}\in \Pi }U_{I}\left(\alpha ,\rho^{*}\right)$.

#### 3.3.4. Toy Example

Let us now revisit the example from Section 3.2.4 and expand it for the BCIG. In addition to the previously given constant values for edges $e_{1},e_{2}$, we define the following: $\alpha_{1}^{*}=\kappa_{1}^{*}=3,\beta_{1}^{*}=20,\alpha_{2}^{*}=\kappa_{2}^{*}=2$ and $\beta_{2}^{*}=15$. Moreover, the Investigator has the following belief: the Attacker’s type is $\theta_{1}$ with probability 0.6 and $\theta_{2}$ with probability 0.4. Using Equations (7) and (8), we populate Table 3. Finally, using the Harsanyi transformation [25], we transform Table 3 to normal form as shown in Table 3. The pure strategies for the Investigator are the same as in Section 3.2.4, while for the Attacker are $\left\{e_{1}e_{1},e_{1}e_{2},e_{2}e_{1},e_{2}e_{2}\right\}$. By solving the game, we acquire the following Equilibrium for mixed strategies: α={0.01794872,0,0.98205128,0} and ρ={0.53076923,0.46923077}. This means that the Attacker’s optimal RAP is to mix over the strategies $\left\{e_{1},e_{1}\right\}$ and $\left\{e_{2},e_{1}\right\}$, i.e., play $e_{1}$ with probability 0.01794872 and $e_{2}$ with probability 0.98205128 when $\theta =\theta_{1}$ and always play $e_{1}$ when $\theta =\theta_{2}$. Thus, the IRP is to play $e_{1}$ with probability 0.53076923 and $e_{2}$ with probability 0.46923077.

## 4. Case Study

In this section, we present a case study that consists of 33 TTPs from the MITRE ATT&CK knowledge base and their corresponding benefit and cost values collected using CVSS and interviews with cyber-security practitioners. We use the Tactics, Techniques, and Procedures (TTPs) to create a representative graph of incidents where we evaluate the efficiency of IRP and two other investigative methods, namely Uniform and Common Sense Strategy (CSS), against three types of Attackers, a strategic, a uniform and a common sense.

### 4.1. Action Space

**Figure 1:**
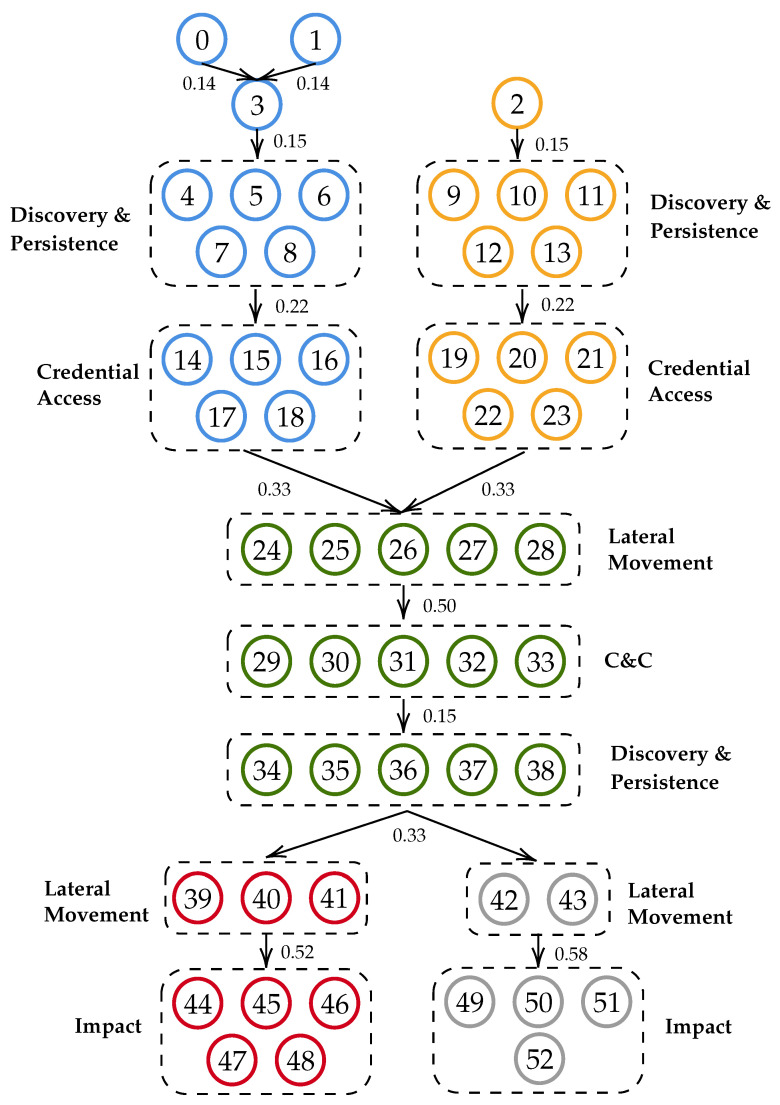
Use case graph utilizing nodes of Table 4.

To evaluate IRP, we use a subset of some of the most popular TTPs [26] available in the MITRE ATT&CK Enterprise matrix to create the graph of Figure 1. The graph is a small-scale representation of a corporate network with multiple hosts and multiple attack actions (TTPs) available at each host. Each attack action is represented by an edge on the graph, while each decision point, i.e., the point in time when players decide their next move, is represented by a node. As this is a multi-host environment, we use different colours to represent different hosts of the network. In this way, decision points (i.e., nodes) that belong to the same host are outlined with the same colour. Edges connect decision points in a logical way, for instance, for the Attacker to move from any Discovery & Persistence node (Nodes 4–8) of the blue host to any Lateral Movement node of the green host (Nodes 24–28), he/she needs to first acquire the required credentials through any Credential Access node (Nodes 14–18). This is because acquiring valid credentials is a requirement for all the Lateral Movement TTPs used in this case study by the Attacker to move to the next host.

To simplify the representation of Figure 1, we have organised the nodes that are connected to the same nodes in groups using the Tactics of MITRE ATT&CK. For instance, nodes 4 to 8 hold TTPs that fall under the Discovery & Persistence Tactics and are all connected with nodes 14 to 18, which fall under the Credential Access Tactic. Thus, instead of adding one to one edge from each node to another one, which would lead to 25 edges between those nodes, we simply add one edge between the two groups of nodes. This means that all nodes 4 to 8 are connected with all nodes 14 to 18. Furthermore, for simplicity reasons, when a player is on a decision point, we refer to the available edges on this decision point using the number of the node on which the edge ends. For instance, if a player is on decision point 5 the available edges would be edges 14 to 18, i.e., the edges that lead to nodes 14 to 18. Finally, we use a subset of the TTPs available under the Evasion Tactic of MITRE ATT&CK as the anti-forensic techniques. For instance, for any edge that holds the Standard Application Layer Protocol TTP the available anti-forensic technique is the Data Encrypted TTP, as it allows the data transported through the C&C channel to be private and even if the Investigator is able to decrypt them it will take a considerable amount of time and resources. Table 4 presents the TTP used for the edges (and nodes) of the graph, while Table 5 presents the anti-forensic technique assigned for each edge.

### 4.2. Benefit and Cost Parameters

Table 6 presents the benefit $\beta_{i}$ and cost $\kappa_{i},\alpha_{i}$ values for each TTP as well as the corresponding values $\beta^{*},\alpha^{*},\kappa^{*}$ for each anti-forensic TTP. Here, we have chosen for simplicity reasons to allow equal cost values for the Attacker and the Investigators.

As previously explained in Section 3, the benefit value of each edge represents the *impact* that the corresponding TTP would have to the organisation if taken by the Attacker. For this reason, in this paper, we use the Common Vulnerability Scoring System (CVSS) to collect these values (https://nvd.nist.gov/vuln-metrics/cvss/v3-calculator, accessed on 4 August 2021). CVSS is a publicly available standard that allows professionals to produce a numerical score that reflects the severity of a vulnerability based on its characteristics. Although CVSS has three available metrics, Base, Temporal, and Environmental Score, in this paper, we only use the Base Score metric. Specifically, we utilise the Base Score metric to input characteristics for each TTP, such as its impact on the confidentiality, integrity and availability (CIA) triad or the required privileges, to calculate its benefit value.

As there is no standardized metric or framework that can be used to calculate the cost value of each TTP (and thus each edge) we performed a two-phase set of interviews with six experienced cyber-security practitioners to collect the required values. Background information and experience of each interviewee can be found in Table 7. During the first phase of interviews, the practitioners were asked which criteria they believe were appropriate for the valuation of the cost of an attack action as a representation of the time need for its analysis. The criteria that were identified through this first phase were: (i) the type of the analysis, i.e., level of automation through tools, and (ii) the complexity of the analysis, i.e., technical difficulty, amount of required data and data sources to be analysed. Both of those criteria affect the amount of time that is required to analyse an attack action and reveal its corresponding evidence. At the second phase of the interviews, each practitioner was asked to rate the cost of each TTP in a scale of one to ten based on the previously identified criteria. For instance, if the Standard Application Layer Protocol TTP has a cost of 6 while the Registry Run Keys/Startup Folder has a cost of 3, because the former (i) requires the analysis of a large amount of network traffic data while the latter only requires the analysis of the registry and (ii) includes tasks of higher difficulty such as decryption and decoding. To allow the reader to get a better understanding of the cost values, we provide an estimate correspondence between cost values and analysis time in minutes in Table 6.

### 4.3. Attacker Type Probabilities

As explained in Section 3.3, at each individual BCIG, the Attacker is of type $\theta_{2}$, i.e., uses anti-forensic action, with probability *p* and $\theta_{1}$, i.e., does not use an anti-forensic action, with probability 1−p. Thus, we need to calculate the probability of the Attacker using anti-forensic at each decision point of the graph. However, every decision point has a set of child edges, which all have their individual probability to be used along their corresponding anti-forensic technique. Thus, the overall probability of using an anti-forensic technique in the decision point would be equal to the probability of undertaking any of the connected edges with an anti-forensic technique, i.e., the union of those probabilities.

To acquire this probability, we firstly need to calculate the probability of the Attacker using an anti-forensic TTP for each individual edge connected to the decision point. To do so, we use the MITRE ATT&CK STIX Repository (https://github.com/mitre/cti, accessed on 4 August 2021)). The repository uses the STIX 2.0 (https://oasis-open.github.io, accessed on 4 August 2021)) standardized language to hold all the information collected and processed by MITRE through publicly available incident reports regarding adversarial TTPs. Specifically, each TTP, Threat Group, Software etc. has been assigned to an STIX Domain Object (SDO), which contains several information fields, such as name, reference IDs and other object specific fields. For instance, an SDO for a specific TTP may contain fields such as required privileges, which platform and application it may be used against etc. The relationships between these SDOs are represented using STIX Relationship Objects (SROs), which have three mandatory fields: relationship type, source ref and target ref. For example, if a TTP is being used by a Threat Group, there will be an SRO that connects the SDO of this TTP with the Threat Group’s SDO that will also contain an additional field holding the name or URL of the relevant reports.

To query the repository for all the available reports and URLs for each selected TTP, we use the python-stix2 (https://github.com/oasis-open/cti-python-stix2, accessed on 4 August 2021)) library. Then, we use python dictionaryto hold the results so that each key of the dictionary corresponds to a TTP and its value holds a list of the reports and URLs connected to this TTP. Finally, we correlate the available reports for each TTP with the reports of its anti-forensic TTP in order to calculate the probability of them being used together. Then, we take the union of the probabilities of each edge connected to the decision point to calculate *p*. As the events are not mutually exclusive, we also need to subtract the probabilities of their intersection, which we calculate from the repository in a similar way as *p*. The produced anti-forensic probability for each decision node can be found in Figure 1.

### 4.4. Simulations

In order to evaluate the efficiency of Investigator’s Optimal Randomised Plan (IRP), we compare it with two other investigative methods, the Uniform method and the Common Sense Strategy (CSS). The Investigator using the Uniform method selects an edge according to a uniform distribution while the Investigator using the CSS selects an edge using the benefit value as the sole criterion. Specifically, the CSS Investigator acts as a rational player that is motivated by the benefit of each edge and thus selects each edge $e_{i}$ with probability $\beta_{i}/\sum_{e_{k}\in E}\beta_{k}$. All three types of Investigators are evaluated against three Attacker types, a strategic, a Uniform and a common sense. The strategic Attacker plays an optimal RAP as described in Definition 2, while the uniform Attacker uses a uniform distribution to select edges similarly to the Uniform investigative method. Finally, the common sense Attacker uses the benefit value of each edge as the criteria similarly to the CSS Investigator in order to create a weighted probability distribution as an investigative method.

We simulate a high number of incidents, where each incident is composed of a set of edges and decision points (nodes) of Figure 1. At each decision point, each player chooses an edge using their corresponding probability distribution, which in the case of the Attacker and IRP Investigator comes from solving a BCIG game. To initiate an incident, we allow the Attacker to pick an initial decision point from a uniform distribution of starting points (nodes 0, 1 and 2) and traverse the graph based on the chosen edges and the allowed movements until he/she reaches the target nodes (nodes 44–52). Then, all players make a choice for each node in the incident and accumulate their collected payoff based on the choice of the Attacker. The high number of incidents has been selected to emulate the usage of the strategies by a cyber-security company that employs a large number of Investigators working on a large number of cases.

### 4.5. Results and Discussion

We compare the performance of all three investigative methods against three types of Attackers on two different levels: (i) accumulated payoff from all edges of the investigated incidents and (ii) accumulated payoff per TTP tactic.

**Figure 2:**
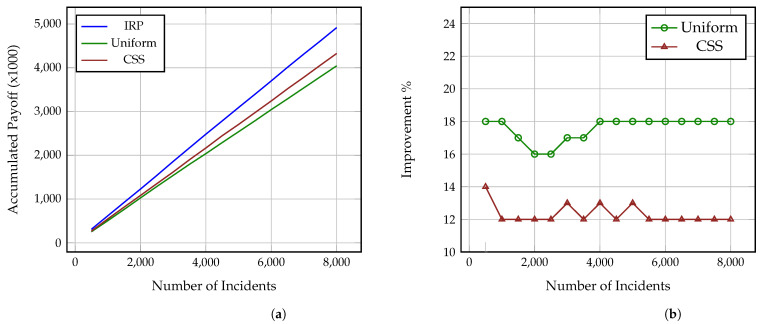
Performance comparison of IRP, Uniform and CSS on N = 8000 simulated incidents against Strategic Attacker. (**a**) Accumulated payoff, (**b**) Percentage of IRP improvement.

**Figure 3:**
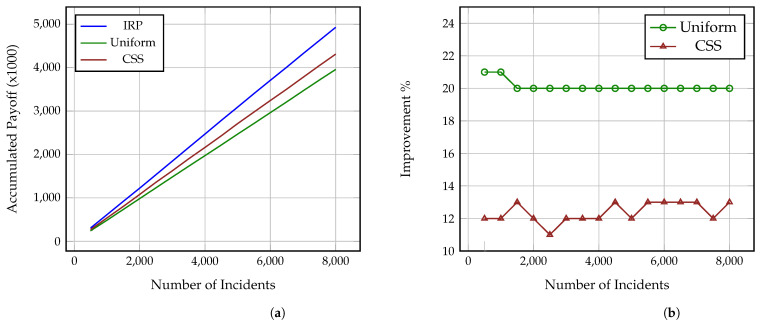
Performance comparison of IRP, Uniform and CSS on N = 8000 simulated incidents against CSS Attacker. (**a**) Accumulated payoff, (**b**) Percentage of IRP improvement.

**Figure 4:**
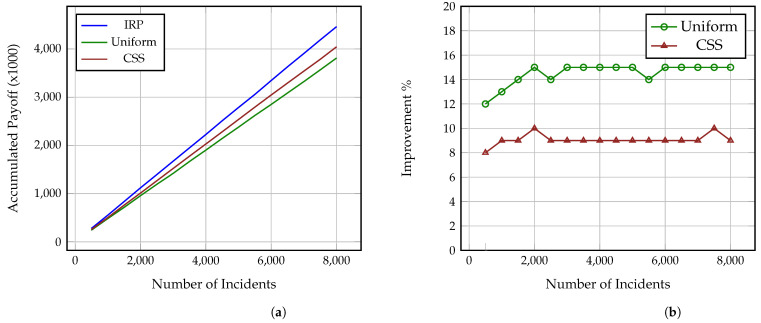
Performance comparison of IRP, Uniform and CSS on N = 8000 simulated incidents against Uniform Attacker. (**a**) Accumulated payoff, (**b**) Percentage of IRP improvement.

Figure 2a, Figure 3a and Figure 4a present how the accumulated payoff of the Investigators using IRP, Uniform and CSS increases as the number of investigated incidents grows. Each figure corresponds to one of the aforementioned Attackers, i.e., strategic, common sense and uniform, as explained in Section 4.4. As it is shown, the Investigator using IRP steadily outperforms both the Uniform and CSS Investigators against all three Attackers and while, at a low number of incidents, the difference in the collected payoff is small, after 8000 incidents, the IRP Investigator has collected a payoff between 4500–5000 while the CSS and Uniform Investigators have collected payoffs between 4000–4300 and 3800–4000, accordingly. Thus, as the number of incidents increases, the gap difference between the accumulated payoff from the three strategies increases as well. This also means that the more complex the incidents under investigation are, i.e., include more impactful and greater number of attack actions, the more significant this difference becomes, even at a much lower number of incidents. It is also worth noting here that these values derive from Formulas (7) and (8) and so represent both the gained benefit, i.e., decrease of the adversarial impact on the attacked organisation, and the conserved cost (time) of the Investigator. Thus, an increase of the payoff is caused by an increase of the accumulated benefit and decrease of the spent analysis time.

Similarly, Figure 2, Figure 3 and Figure 4 show the percentage of performance improvement that IRP offers compared to CSS and Uniform Investigators against each of the three Attackers. Specifically, the improvement provided by IRP on the Uniform Investigator ranges between: (i) 16% and 18% for the strategic Attacker, (ii) 20% and 21% for the common sense Attacker, and (iii) 12% and 15% for the uniform Attacker. Similarly, the improvement achieved by IRP on the CSS Investigator ranges between: (i) 12% and 14% for the strategic Attacker, (ii) 11% and 13% for the common sense Attacker, and (iii) 8% and 10% for the uniform Attacker. This reinforces the previous remark on how the long term usage of IRP on complex multi-stage incidents can provide a significant amount of collected payoff to an organisation, especially if utilised by all the employed investigators and analysts. This improvement directly translates to: (i) conserved investigation time, which can be used for other investigations, and (ii) decrease of the adversarial impact on the victim organisations through the reveal of the performed attack actions.

**Figure 5:**
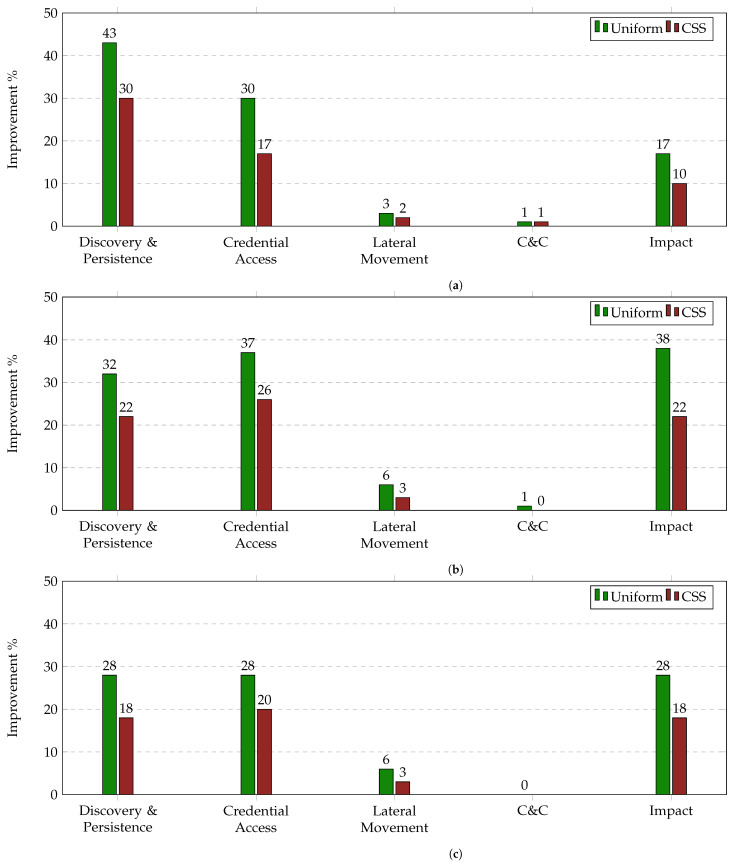
Average improvement of IRP on Uniform and CSS per TTP Tactic on N = 8000 simulated incidents. (**a**) Percentage of IRP improvement per TTP category for Strategic Attacker, (**b**) Percentage of IRP improvement per TTP category for CSS Attacker, (**c**) Percentage of IRP improvement per TTP category for Uniform Attacker

Figure 5a–c present the average improvement of IRP on Uniform and CSS per TTP Tactic across 8000 incidents. This allows us to get a deeper understanding on how the number of available edges at each decision point and the variety of their cost and benefit values affect the performance of each strategy.

As shown, the highest improvement on both the Uniform and CSS method across all three Attackers is achieved in the Discovery & Persistence, Credential Access and Impact categories while the lowest is achieved in the Command and Control (C&C) and Lateral Movement categories. By correlating these results with the information provided by Table 4 and Table 6, we can observe that the higher the variety and number of available edges at a decision point, the higher the enhancement of the performance. Specifically, the Discovery & Persistence Tactic includes in all cases 5 different TTPs with benefit values that range from 33 to 61 and cost values that range from 3 to 5. On the contrary, the C&C Tactic includes 2 different TTPs, i.e., the Standard Application Layer Protocol and the Custom C&C Protocol, that have identical benefit values and cost values of 6 and 8. The reuse of those TTPs is caused by: (i) the fact that they may represent a number of different protocols, and (ii) the size limitations of this case study. Thus, taking in consideration that in reality the number of available edges at each decision point would be much higher that the one used in this case study, we should expect a higher percentage of overall performance improvement compared to the numbers presented in Figure 2 as the variety of the edges increases in all categories.

## 5. Limitations and Future Work

In a real-world scenario, the proposed method can be utilised as a guide by the Investigator to navigate step by step the analysis of a multi-stage cyber incident. Specifically, if we consider the incident as a set of steps, i.e., attack actions, the investigation is a set of forensic actions too, i.e., inspections of the attack actions. In this way, IRP can be used at each step to generate an optimal strategy and, thus, inspection action for the Investigator, in a similar way that our previous work DISCLOSE [2], was utilised. This allows the Investigator to incorporate in his/her decision process the strategic nature of the Attacker but also the cost and benefit factors. Moreover, IRP could be part of a more complex decision support system, expanded with further parameters and combined with other optimisation methods in order to increase the quality of the suggestions provided to the Investigator. For real life application of IRP, the cost and benefit values could be calculated per organisation using: (i) information regarding the available assets, such as their role and importance, and (ii) data from past incident “tickets”, such as the required analysis time.

Regarding the evaluation of the efficiency of IRP, in this work we used a realistic case study, but in the future we aim to expand our evaluation against graphs with greater TTP variety, both in terms of benefit and cost values and number of edges available per decision point. This will allow us to investigate the extent to which the growth of the complexity of the graph in general but also per decision point will improve IRP. Similarly, we would like to evaluate the optimality of IRP’s suggestions and the support that it can offer to Investigators using participants instead of simulations.

Moreover, we would like to extend IRP by incorporating a budget parameter for both the Attacker and the Investigator that will allow the optimisation of the whole incident path by taking in consideration future edges and using a discount factor. Finally, in this work, we assumed for simplicity reasons that only one anti-forensic technique is available per edge but, in reality, there will be more than one option for the Attacker. IRP can be extended to take this in consideration by assuming more than one Attacker types at each decision point, i.e., each individual game.

## 6. Conclusions

As adversaries become more sophisticated and stealthier, the use of anti-forensic techniques for the minimisation of the likelihood of their detection is increased. As a result, cyber investigations become more complex and challenging and investigators need support methods that will allow them to increase their efficiency against strategic adversaries. To this end, in this paper, we have modeled a multi-stage cyber attack as a set of multiple Bayesian games of incomplete information that take place on a graph of attack actions. We define two Attacker types, one that uses anti-forensic techniques and one that does not, based on a probabilistic distribution that is calculated through past incident reports. In this way, the Investigator is able to create an optimal investigative strategy, called Investigator’s Optimal Randomised Plan (IRP), which takes in consideration the uncertainty regarding the Attacker’s type as well as the potential benefit and cost values of each action.

We evaluate IRP against two other investigative methods, namely Uniform and CSS, using a case study of 33 TTP from MITRE ATT&CK and three different Attacker’s methodologies. To this end, we collect data from the MITRE ATT&CK STIX repository, Common Vulnerability Scoring System (CVSS) and six interviews with cyber-security practitioners. According to our results, IRP outperforms the Uniform method by 18% and CSS method by 14% in terms of accumulated payoff. Moreover, our evaluation suggests that the improvement achieved by IRP is even higher at nodes with higher number of edges and greater diversity of benefit and cost values.

## Figures and Tables

**Table 1:** List of symbols.

Symbol	Description
Constants	
$\beta_{i}$	benefit associated with edge $e_{i}$ for both players
$\alpha_{i}$	Attacker’s cost for edge $e_{i}$
$\kappa_{i}$	Investigator’s cost for edge $e_{i}$
$\beta^{*}$	benefit associated an anti-forensic technique
$\alpha^{*}$	Attacker’s cost for an anti-forensic technique
$\kappa^{*}$	Investigator’s cost for an anti-forensic technique
Functions and variables	
E	set of edges available on any decision point
$e_{i}$	*i*th edge on any decision point
$U_{A}$	payoff function for Attacker
$U_{I}$	payoff function for Investigator
θ	Attacker’s type
ρ	mixed strategy for the Investigator
α	mixed strategy for the Attacker

**Table 2:** Payoffs for example CFG.

		Investigator	
		$e_{1}$	$e_{2}$
Attacker	$e_{1}$	−8, 62	62, −7
	$e_{2}$	53, −8	−7, 53

**Table 3:** Illustrative example of BCFG. (**a**) Payoffs table for BCFG before transformation, (**b**) Payoff table after Harsanyi transformation.

a			
Attacker		Investigator	
		$e_{1}$	$e_{2}$
	Type $\theta_{1}$		
	$e_{1}$	−8, 62	62, −7
	$e_{2}$	53, −8	−7, 53
	Type $\theta_{2}$		
	$e_{1}$	−11, 79	79, −9
	$e_{2}$	66, −11	−9, 66
**b**			
Attacker		Investigator	
	$\theta_{1},\theta_{2}$	$e_{1}$	$e_{2}$
	$e_{1},e_{1}$	−9.2, 68.8	68.8, −7.8
	$e_{1},e_{2}$	21.6, 32.8	33.6, 22.2
	$e_{2},e_{1}$	27.4, 26.8	27.4, 28.2
	$e_{2},e_{2}$	58.2, −9.2	−7.8, 58.2

**Table 4:** Technique contained in each node of graph.

Nodes	Technique
0, 27, 42	Spearphishing Attachment
28, 43	Spearphishing Link
1	Replication Through Removable Media
2	External Remote Services
3	User Execution
4, 9, 34	System Service Discovery
5, 10, 35	System Information Discovery
6, 11, 36	Registry Run Keys/Startup Folder
7, 12, 37	Modify Existing Service
8, 13, 38	New Service
14, 19	Credential Dumping
15, 20	Credentials In Files
18, 23, 26, 41	Brute Force
16, 21, 52	Create Account
24, 39	Pass the Hash
25, 40	Windows Remote Management
29, 31, 33	Standard Application Layer Protocol
30, 32	Custom C&C Protocol
46, 49	Data Encrypted for Impact
44	Data from Local System
45	Data from Network Shared Drive
50	Exfiltration Over Alternative Protocol with Data from Local System
47	Exfiltration Over C&C Channel with Data from Local System
51	Exfiltration Over C&C Channel with Data from Network Shared Drive
48	Exfiltration Over Alternative Protocol with Data from Network Shared Drive

**Table 5:** Anti-forensic actions per node in the Use Case.

Nodes	Technique
0, 27, 28, 42, 43	Obfuscated Files or Information
1,3,6, 8, 11, 13, 36, 38	Modify Registry
2, 16, 21, 24, 25, 39, 40,52	Valid Accounts
4, 5, 7, 9, 10, 12, 14, 15, 17, 18, 19, 20, 22, 23, 26, 34, 35, 37, 41	Indicator Removal on Host
29, 30, 31, 32, 33, 45, 46, 47, 48, 49, 50, 51	Data Encrypted

**Table 6:** Cost and benefit values for each Technique used in the case study.

Technique	Benefit	Cost (Score)	Cost (Min.)
Node Techniques			
Spearphishing Attachment	48	5	20–30
Spearphishing Link	48	5	20–30
Replication Through Removable Media	36	5	20–30
External Remote Services	74	7	60–120
User Execution	47	3	10–15
System Service Discovery	33	5	20–30
System Information Discovery	33	5	20–30
Network Share Discovery	33	5	20–30
Registry Run Keys/Startup Folder	55	3	10–15
Modify Existing Service	61	3	10–15
New Service	61	3	10–15
Credential Dumping	47	8	120–240
Credentials In Files	47	5	20–30
Brute Force	72	5	20–30
Create Account	63	6	30–60
Pass the Hash	78	7	60–120
Windows Remote Management	78	7	60–120
Standard Application Layer Protocol	63	7	60–120
Custom C&C Protocol	63	8	120–240
Data Encrypted for Impact	57	6	30–60
Data from Local System	71	6	30–60
Data from Network Shared Drive	71	5	20–30
Exfiltration Over Alternative Protocol with Data from Local System	102	10	360+
Exfiltration Over C&C Channel with Data from Local System	102	10	360+
Exfiltration Over C&C Channel with Data from Network Shared Drive	102	10	360+
Exfiltration Over Alternative Protocol with Data from Network Shared Drive	102	10	360+
Anti-forensic Techniques			
Obfuscated Files or Information	60	7	60–120
Modify Registry	55	3	10–15
Valid Accounts	47	5	20–30
Indicator Removal on Host	40	5	20–30
Data Encrypted	60	7	60–120

**Table 7:** Background information of experts interviewed for gathering the cost values $\alpha_{i},\kappa_{i}$.

#	Job Title	Main Duties	Experience (Years)
1	Security Operations Advisor	Strategic advisory on SecOps	12
2	Digital Forensics Expert	DFI chapter leader for Advisory firm	20
3	Senior Lecturer in Incident Response & Digital forensics	Research, Consulting & Teaching in Incident Reponse/OWASP Dorset Chapter Leader	13
4	Enterprise Security Architect	Review/deploy of Security Architectures, PoC of identified solutions, Security controls mapping	14
5	Senior Red Team Analyst	Intelligence-led and objectives-focused adversarial emulations. OSINT, social engineering, C&C, post-exploitation, physical attacks	10
6	Associate security consultant	Penetration testing, reporting and client scoping	2

## Data Availability

The data can be found at: https://github.com/isec-greenwich, accessed on 4 August 2021).

## Abbreviations

The following abbreviations are used in this manuscript:

APTs	Advanced Persistent Threats
TTPs	Tactics, Techniques, and Procedures
STIX	Structured Threat Information Expression
CVSS	Common Vulnerability Scoring System
NCIG	Nash Cyber Investigation Game
NE	Nash Equilibrium
IRP	Investigator’s Optimal Randomised Plan
BCIG	Bayesian Cyber Investigation Game
BNE	Bayesian Nash Equilibrium
CSS	Common Sense Strategy
SDO	STIX Domain Object
SRO	STIX Relationship Objects
